# Malaria and Early Life Immunity: Competence in Context

**DOI:** 10.3389/fimmu.2021.634749

**Published:** 2021-02-19

**Authors:** Perri C. Callaway, Lila A. Farrington, Margaret E. Feeney

**Affiliations:** ^1^Infectious Diseases and Immunity Graduate Group, University of California, Berkeley, Berkeley, CA, United States; ^2^Department of Medicine, University of California, San Francisco, San Francisco, CA, United States; ^3^Department of Pediatrics, University of California, San Francisco, San Francisco, CA, United States

**Keywords:** fetal immunity, neonatal immunity, malaria, plasmodium, neonatal vaccination

## Abstract

Childhood vaccines have been the cornerstone tool of public health over the past century. A major barrier to neonatal vaccination is the “immaturity” of the infant immune system and the inefficiency of conventional vaccine approaches at inducing immunity at birth. While much of the literature on fetal and neonatal immunity has focused on the early life propensity toward immune tolerance, recent studies indicate that the fetus is more immunologically capable than previously thought, and can, in some circumstances, mount adaptive B and T cell responses to perinatal pathogens *in utero*. Although significant hurdles remain before these findings can be translated into vaccines and other protective strategies, they should lend optimism to the prospect that neonatal and even fetal vaccination is achievable. Next steps toward this goal should include efforts to define the conditions for optimal stimulation of infant immune responses, including antigen timing, dose, and route of delivery, as well as antigen presentation pathways and co-stimulatory requirements. A better understanding of these factors will enable optimal deployment of vaccines against malaria and other pathogens to protect infants during their period of greatest vulnerability.

## Neither Man nor Mouse: Immune Competence in the Infant

The human infant has long been considered immunologically immature, or deficient, when judged against the yardstick of the adult immune system. Indeed, the fetus and newborn are vulnerable to severe disease and high morbidity from numerous pathogens that cause only mild disease in older hosts. It had previously been believed, based largely on extrapolation from mouse models of immune development, that the tolerogenic intrauterine environment is incompatible with the priming of fetal T cells or results in marked polarization of CD4 T cells toward a Th2 response over a Th1 response. However, it is now appreciated that human fetal immune development differs quite markedly from that of the mouse. Neonatal mice are profoundly immunodeficient—indeed, migration of murine T cells from the thymus begins only after birth. In contrast, during human gestation, T cells appear in the fetal liver at week 10 and T cell zones can be seen in the spleen during week 18 (1). The recent development of single-cell analysis tools has led to a remarkable burst of progress in the study of fetal and infant immune cell populations, and has revealed a surprising degree of immune competence at, and even before, full-term gestation (2–5).

Placental malaria offers a valuable model to examine the response to pathogen-derived antigens *in utero*, and findings from this model bear relevance to other parasitic pathogens. Annually, more than 125 million pregnancies occur in areas where malaria is endemic (6), and 25% of pregnancies in sub-Saharan Africa are complicated by malaria (7). While true congenital infection with malaria parasites is rare, malaria antigens gain access to the fetal circulation after crossing the placenta, and studies of cord blood from malaria-exposed neonates have demonstrated an impact on numerous immune cell populations (2, 8, 9). Thus, understanding the immunological implications of placental malaria exposure is of paramount importance to public health.

## Learning to Adapt: T and B Cells

Not surprisingly, most T and B cells in umbilical cord blood are naïve in phenotype, reflecting the relative lack of antigen experience during intrauterine life. During early and mid-gestation, human fetal T cells are inclined toward tolerance, as has been reviewed elsewhere (10, 11). Upon encounter with non-self-antigens, naïve CD4 T cells in the mid-gestation fetus preferentially differentiate into FoxP3+ regulatory T-cells that, along with other regulatory populations, can actively suppress T cell activation and cytokine production (12–14). However, as the fetus approaches term, it must balance the demands of maternal tolerance with the need to mount an effector T cell response against potential pathogens encountered after birth. It was recently shown that even in the absence of intrauterine pathogen exposure, a sizeable subset of CD4 T cells in cord blood exhibit effector-memory differentiation and can produce both Th1 and Th2 cytokines (15). Indeed, TNFα and IFNγ production by fetal T cells that are alloreactive to maternal antigens may contribute to preterm labor (16). Whether fetal Th1 responses to pathogens such as malaria may contribute to preterm birth or poor growth *in utero* has not been adequately investigated.

Until recently, relatively few studies have applied single-cell analysis techniques to characterize the human T cell response to pathogen-derived antigens encountered *in utero*. In aggregate, these studies suggest that despite the numerous mechanisms enforcing fetal and neonatal tolerance, the fetus is capable of mounting robust T cell responses under particular conditions. Congenital viral infections such as CMV result in expansion and differentiation of virus-specific CD8 T cells that produce IFNγ, TNFα, and perform perforin-mediated cytolysis (17). Recently, we showed that in the setting of placental malaria, CD4 and CD8 T cells primed *in utero* demonstrate effector-memory differentiation, inflammatory cytokine production, and robust malaria antigen-specific T cell proliferation. These effector populations were associated with protection from both *P. falciparum* infection and symptomatic malaria during the first 2 years of life (2). The most profound effector T cell differentiation was observed in infants born to mothers with active placental malaria at the time of birth, possibly suggesting late gestation exposure (2). However, T cell responses were not observed in all exposed infants in this study, and other investigators have observed that *in utero P. falciparum* exposure tolerized fetal T cells in a subset of exposed infants (18). The timing, duration, and quantity of malaria antigen exposure (influenced by intermittent preventive therapy with antimalarials), as well as the degree of associated placental inflammation, may play a large role in influencing the balance between fetal T cell tolerance vs. effector differentiation.

Some evidence indicates that fetal B cells can also be primed *in utero*. Malaria-specific antibodies of the IgM class (which cannot cross the placenta) have been detected in the cord blood of malaria-exposed infants as early as 22 weeks gestation (8, 9, 19) and surprisingly, class-switching of malaria-specific B cells from IgM to IgG occurs in some infants prior to delivery (8). These findings raise the possibility that fetal B cells could be sensitized by maternal malaria vaccination, as has been reported with maternal tetanus and influenza vaccination (20, 21).

Together, these data indicate that the essential machinery for generation of robust T and B cell responses to pathogen-derived antigens is present during fetal life. They further suggest that the mechanisms that curb T cell inflammation *in utero* are not entirely cell-intrinsic, but also relate to extrinsic factors such as a lack of sufficient activating or co-stimulatory signals from antigen presenting cells (APCs) or from a tolerogenic cytokine environment. A better understanding of the conditions (e.g., timing, antigen load) that foster the priming and development of functionally competent pathogen-specific T cells (while avoiding induction of pathogen tolerance) could be of fundamental importance for efforts to develop vaccines that are optimally immunogenic in infancy.

## APCs: Presentation Matters

**Figure 1:**
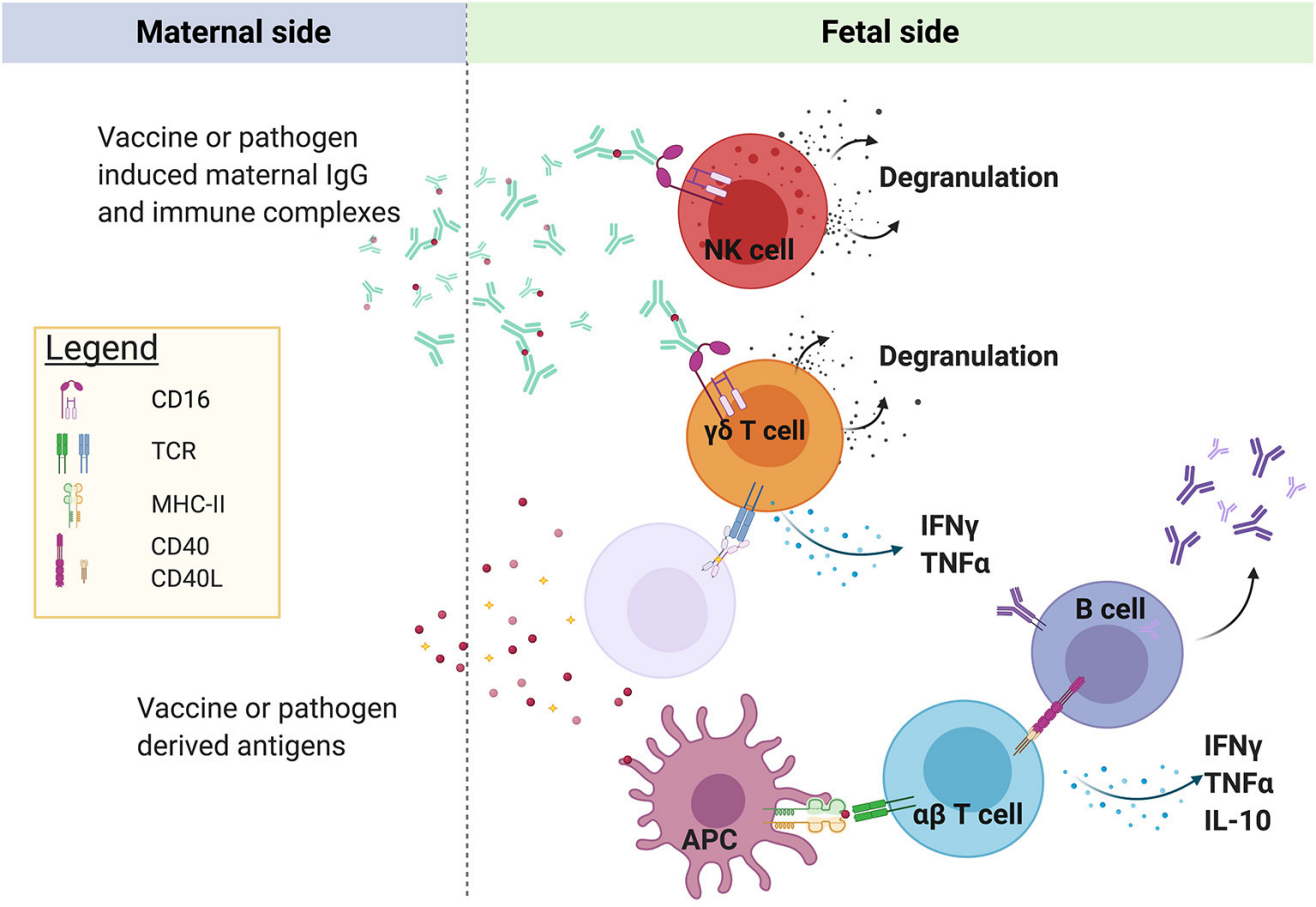
Maternal-origin IgG is transported across the syncytiotrophoblast barrier of the placenta to the fetus via FcRn, possibly in the form of immune complexes. In areas of placental villous denudement or necrosis, unbound plasmodial antigen may also cross into the fetal circulation. *P. falciparum* antigens have been shown to prime fetal αβ T cells and B cells, the location and identity of the antigen-presenting cells remain unknown, but could include fetal Hofbauer cells, dendritic cells, or γδ T cells. Semi-innate Vγ9Vδ2 T cells can be directly activated by plasmodial-derived phosphoantigens via butyrophilin2a1 and butyrophilin3a1, even in the absence of prior antigen exposure. In addition, fetal lymphocytes expressing CD16/FcRγIIIa, including NK cells and possibly γδ T cells, may be activated by maternal IgG bound to antigen. Created with BioRender.com.

The maternal and fetal blood supply are separated by a single multinucleated cell layer termed the syncytiotrophoblast. Once malaria antigens or immune complexes cross the syncytiotrophoblast barrier, it is not clear where, how, and by “whom” (i.e., what cell type) they are taken up, processed, and presented to lymphocytes (Figure 1). This is a critical question, as APCs are key orchestrators of the immune response and play a paramount role in the initiation and regulation of adaptive immune responses through priming of antigen-specific T cells. Murine data indicate that neonatal T cells are extremely sensitive to the conditions of antigen presentation at priming, and small differences in the dose of antigen (22), type of APC (22, 23), and intensity of costimulation (22–24) strongly influence the efficacy of the ensuing T cell response. Given the many shortcomings of the neonatal mouse model (25), further studies are needed to confirm the relevance of these findings in human infants.

In adults, myeloid-lineage cells such as dendritic cells (DCs) and monocytes play a principal role in antigen presentation, although activated CD4 T and B cells also upregulate HLA-DR and can present antigen (26–28). In the fetus and neonate, dendritic cells and monocytes are both relatively inefficient in their ability to prime adaptive immune responses due to their reduced expression of MHC-II, co-stimulatory molecules, and Th1-polarizing cytokines (29–31). In particular, neonatal DC production of IL-12p70, the key cytokine required for Th1 polarization, is markedly reduced due to epigenetic regulation of the gene encoding its p35 subunit (29, 31–33). Th1 cytokine production by fetal DCs may be further inhibited by expression of arginase-2 (4). Fetal monocytes are also inefficient in their upregulation of costimulatory and antigen presentation machinery in response to IFNγ (34) despite enhanced sensitivity to inflammatory cytokines and increased expression of the IL6 receptor. Instead, inflammatory cytokines activate non-canonical signaling pathways in fetal monocytes, leading to upregulation of genes involved in the primitive antimicrobial response (34). This is likely a strategy to prevent activation of a potentially harmful anti-maternal adaptive response, which may trigger preterm labor and fetal expulsion (16).

It is possible that alternative cell populations may play a particularly important role in antigen presentation during fetal life. Macrophages termed Hofbauer cells reside within the placental villous stroma and express multiple Fc receptors, making them well-suited to a role in immune surveillance. Hofbauer cells are increased in placental malaria (35), but whether they play a role in antigen uptake is unclear. In an *ex vivo* human placental perfusion model, upon transplacental transfer immune-complexed MSP-1 was observed in the fetal villous stroma where it predominantly co-localized with fetal endothelial cells, not Hofbauer cells (36). Moreover, a recent and very detailed phenotypic analysis by Thomas et al. found that fetal-origin macrophages do not express HLA-DR (37). It is possible that non-myeloid cells, including non-classical lymphocytes, contribute to the induction of adaptive immune responses *in utero*. In adults, the Vγ9Vδ2 subset of γδ T cells (discussed in detail below) exhibit robust antigen presentation capabilities upon activation (38, 39) and can induce proliferation and differentiation of naïve αβ T cells comparable to that of mature DCs. Vγ9Vδ2 cells are highly prevalent during the second trimester (40), and their potential role in antigen presentation during fetal life merits further investigation.

## Innate Ability: NK Cells and Non-classical Lymphocytes

Innate immune populations may play a particularly important role in protecting the fetus and infant when little or no immunologic memory exists. In particular, semi-innate γδ T cells have several qualities that make them uniquely suited to protection of the fetus and infant. Unlike conventional αβ T cells, γδ T cells recognize conserved ligands and exhibit rapid, innate-like effector functions, including degranulation and cytokine production (41). This effector response is not dependent on prior antigen exposure nor on priming by dendritic cells, which are functionally immature in the fetus. γδ T cells are highly conserved across vertebrate species and are the first T cells to develop in the human fetus. It has been hypothesized that the primary selective advantage driving their remarkable conservation is their role in neonatal protection (39). Supporting this hypothesis, γδ T cells are required for protection of young, but not mature, mice in models of parasitic infection (42).

During malaria infection, a specialized subset of γδ T cells, defined by use of the δ2 and γ9 TCR chains, can act as innate-like effectors that can indirectly recognize phosphoantigens that are produced by the *Plasmodium* apicoplast (43). These Vγ9Vδ2 T cells exhibit intrinsic reactivity to *Plasmodium*, with rapid degranulation and production of IFNγ and TNFα, even in malaria-naïve individuals (44). They are able to kill extracellular merozoites via release of granulysin and inhibit parasite growth *in vitro* (45). Moreover, they have been associated with protection from malaria in human clinical trials (46, 47). In the fetus, this subset has been shown to dominate the γδ T cell repertoire in the second trimester (40). Following *in utero* malaria exposure, cord blood Vγ9Vδ2 T cells are preferentially activated, produce more IFNγ (48), and exhibit greater memory differentiation (49). Thus, Vγ9Vδ2 T cells may be poised to respond to *Plasmodium* infection in the fetus and neonate.

The role of NK cells and other innate lymphoid cells (ILCs) in the fetal and infant immune response to malaria has received little research attention. Fetal NK cells that develop early in gestation can respond robustly to antibody mediated and cytokine-induced stimulation, but respond weakly to HLA-devoid cells and are more susceptible to TGF-β mediated suppression (50). At birth, NK cells are highly responsive to immune complexes and exhibit robust antibody-dependent functions, including IFNγ production and degranulation (51). Higher cord blood frequencies of CD56^dim^CD16^+^ NK cells have been observed following maternal *P. falciparum* infection (52). In light of recent studies demonstrating an association between NK cell antibody dependent cellular cytotoxicity (ADCC) and malaria outcomes in children and adults (53), these findings raise the possibility that NK cells could play an important antimalarial role *in utero* and in the newborn through engagement of transplacentally acquired malaria-specific antibodies.

## Maternofetal Antibody Transfer: Passive and Aggressive?

During gestation, maternal antibodies are transferred across the placenta to the fetus via an active transport mechanism mediated by FcRn, the neonatal Fc receptor. FcRn is expressed on syncytiotrophoblast cells beginning at approximately 13 weeks gestation (54), and selectively transfers IgG. Transfer of IgG increases as pregnancy progresses, rising sharply in the final month of gestation, such that full-term infants have IgG levels that generally exceed those of the mother (54, 55). FcRn-mediated transport is influenced by maternal IgG concentration and saturates in the setting of high total IgG levels.

Maternally derived antibodies are essential for infant protection from some pathogens that are commonly encountered during infancy. In the case of malaria, the importance of this passive maternal antibody transfer is not clear. It has been widely promulgated that transplacental transfer of antimalarial antibodies is responsible for the relatively low incidence of symptomatic malaria during the first 6 months of life. Yet, a thorough review of this literature found scant evidence to support a protective role in infancy, concluding instead that malaria-specific IgG in cord blood merely serves as a biomarker of maternal exposure (56). It should be noted, however, that most studies to address this relationship evaluated antibodies to only a very limited number of malaria antigens, and measured only total IgG, and not individual IgG subclasses.

IgG subclass and glycosylation can both greatly influence the efficiency of transplacental antibody transfer by FcRn (55). The overall transfer efficiency is highest for the IgG1 subclass, followed by IgG4, IgG3, and IgG2. It is notable that IgG3 is transferred to the fetus with low efficiency, as it is the strongest activator of complement via recruitment of C1q, and its potent opsonizing ability has been linked to clinical protection from malaria in adults and children (57–59). IgG3 also binds more strongly to FcγRIIIa, which is important in ADCC and other antibody-dependent functions, than other IgG subclasses (60). Interestingly, a polymorphism in the FcRn-binding domain of IgG3 (H435) has been associated with increased transplacental transfer of malaria-specific IgG3, increased half-life of IgG3 in the infant, and protection from clinical malaria during infancy (61). Given this evidence, it will be important to understand the IgG subclass profile of transplacentally-transferred IgG in malaria-endemic regions (62), as well as the significance of opsonized antigen, complement activation, and FcR-expressing fetal cells in infant protection from malaria. Furthermore, secondary glycan structures, which can vary across pathogen-specific antibody repertoires within an individual (51, 63, 64), have also been shown to modulate the efficiency of transplacental IgG transfer. For example, digalactosylated Fc-glycans bind more strongly to FcRn leading to increased transplacental transfer (51), and they also preferentially bind FcγRIIIa leading to better activation of neonatal NK cells (51). As vaccine adjuvants can influence antibody subclass and glycosylation (65) which in turn influence placental transfer of antibody (55), vaccine design should prioritize adjuvants that enable better transplacental transfer and activation of neonatal effector cells.

In addition to conferring passive immunity, maternofetal antibody transfer may play a role in conveying pathogens and pathogen-derived antigens to the fetus in the form of antigen-antibody complexes. While some malaria antigens may breach the maternofetal barrier due to focal denudement of the syncytiotrophoblast cell layer, the more common route of transfer is likely via FcRn-mediated active transport of antigen-antibody complexes (8, 36), as supported by experimental evidence from an *ex vivo* placental perfusion model (36). There is also some evidence that FcRn can facilitate congenital infection by mediating transfer of CMV and ZIKV virions in the form of immune complexes (66–68). Hence, maternal immunoglobulins can at times act as a Trojan horse, ferrying pathogen-derived antigens (or even intact pathogens) to the fetus.

## Implications for Vaccination in Pregnancy and Early Infancy

The ultimate goal of better understanding the components of the fetal, neonatal and pregnant immune systems is to enable the translation of this knowledge to vaccine development. Late stage trials are underway for vaccines to prevent placental malaria by inducing maternal antibodies against VAR2CSA, a pregnancy-specific adhesion ligand that is thought to be an important mediator of placental adherence (69). The goal of this vaccine approach is to stimulate maternal immunity and thus prevent placental malaria, sparing the infant from the clinical sequalae of placental insufficiency and inflammation, which include prematurity and poor growth. This distinguishes it from other vaccines targeting pregnant women, for which the rationale is to confer passive IgG-mediated immunity to the infant. Examples of this approach include vaccination against pertussis, influenza, and tetanus, all of which can be safely administered during pregnancy and induce high neutralizing titers of maternal antibodies that are transferred transplacentally and confer protection to the infant during the early months of life (70–73). Immunizing pregnant women against malaria (with RTS,S, whole sporozoites, or other vaccine candidates) could similarly induce transfer of malaria-specific IgG to the infant. However, whether this passive immunization approach would be of value hinges largely upon the still unanswered question of whether malaria-specific IgG antibodies, or a subclass thereof (e.g., IgG3), confer protection in infants. Moreover, it is important to note that maternal immunization can have adverse consequences by interfering with induction of antibodies in the infant. This has been most clearly demonstrated in the case of pertussis and viral pathogens such as measles, where maternal-origin IgG neutralizes vaccine antigen in the infant and thus preempts priming of infant B cells (74–77). However, even non-neutralizing antibodies can inhibit infant antibody production by mechanisms that may include masking of immunogenic epitopes, Fc-mediated engagement of inhibitory receptors, and/or clearance of maternal immune complexes; hence maternal antibody interference may be of concern for malaria. A recent meta-analysis estimated that maternal antibody inhibition results in a reduction of infant antibody responses to a broad range of antigens in common childhood vaccines (78). Interference by maternal-origin IgG has been hypothesized to contribute to the poor immunogenicity of RTS,S in infants (79). Ultimately such concerns may be mitigated by Fc engineering (currently being developed for monoclonal antibody therapies) and tailoring of adjuvants to induce antibodies of the desired subtype and glycosylation characteristics. As it is difficult to predict whether the benefits of maternal antibodies would outweigh the concern for antibody interference, the impact of maternal-origin IgG on infant vaccine responses should be an area of careful empiric investigation as malaria vaccine candidates enter field trials.

Mounting evidence that adaptive T and B cell responses can be primed *in utero* raises the additional question of whether *active immunization* of the fetus may indeed be possible. Such a strategy would be dependent upon delivery of antigen to the fetus in a context that favors priming of antigen-specific fetal T and/or B cells. The demonstration that fetal B and T cells are primed naturally in response to placental malaria in some infants (2, 8) should lend optimism to this prospect. Additionally, it has been shown that fetal T cells are primed in response to maternal influenza vaccination (20). While *in utero* vaccination may seem like a far horizon, these observations provide proof of concept that induction of protective immunity prior to birth may be possible. However, efforts toward fetal immunization would need to address the potential for induction of pathogen-specific tolerance *in utero*, which has been reported in some malaria-exposed infants (18, 80).

## Conclusion

As pregnancy progresses, the fetal immune system gradually evolves from one that is skewed toward tolerance to one that is poised to fight foreign pathogens. The research agenda for translating recent advances in our understanding of fetal and neonatal immunology into vaccines that are safe and immunogenic when administered in early infancy is now coming into focus. Understanding how to best prime adaptive immune responses in the neonate is critical, due to both the increased susceptibility of newborns to infectious diseases and to the increased healthcare contact of this vulnerable population at the time of birth. This will be particularly important in the context of malaria vaccines, as the vast majority of malaria deaths occur in children under the age of five. Recent evidence from studies of infants exposed to perinatal pathogens indicates that fetal B and T cells can be primed and differentiate into effector cells *in utero*, providing grounds for optimism that they may respond to vaccination. In order to harness this intrinsic capability to engender durable antigen-specific memory in neonates, we will need to refine our understanding of which APCs are most able to prime fetal and neonatal immune responses and which adjuvants are best able to target and stimulate these APCs.

## Author Contributions

PC, LF, and MF wrote the manuscript. All authors contributed to the article and approved the submitted version.

## Conflict of Interest

The authors declare that the research was conducted in the absence of any commercial or financial relationships that could be construed as a potential conflict of interest.
